# Cerebral Blood Flow and Oxygen Delivery in Aneurysmal Subarachnoid Hemorrhage: Relation to Neurointensive Care Targets

**DOI:** 10.1007/s12028-022-01496-1

**Published:** 2022-04-21

**Authors:** Teodor Svedung Wettervik, Henrik Engquist, Anders Hånell, Timothy Howells, Elham Rostami, Elisabeth Ronne-Engström, Anders Lewén, Per Enblad

**Affiliations:** 1 Section of Neurosurgery, Department of Neuroscience, Uppsala University, Uppsala, Sweden; 2 Department of Surgical Sciences/Anesthesia and Intensive Care, Uppsala University, Uppsala, Sweden

**Keywords:** Aneurysmal subarachnoid hemorrhage, Cerebral blood flow, Cerebral oxygen delivery (cerebral pressure autoregulation), Clinical outcome, Xenon-enhanced computed tomography

## Abstract

**Background:**

The primary aim was to determine to what extent continuously monitored neurointensive care unit (neuro-ICU) targets predict cerebral blood flow (CBF) and delivery of oxygen (CDO_2_) after aneurysmal subarachnoid hemorrhage. The secondary aim was to determine whether CBF and CDO_2_ were associated with clinical outcome.

**Methods:**

In this observational study, patients with aneurysmal subarachnoid hemorrhage treated at the neuro-ICU in Uppsala, Sweden, from 2012 to 2020 with at least one xenon-enhanced computed tomography (Xe-CT) obtained within the first 14 days post ictus were included. CBF was measured with the Xe-CT and CDO_2_ was calculated based on CBF and arterial oxygen content. Regional cerebral hypoperfusion was defined as CBF < 20 mL/100 g/min, and poor CDO_2_ was defined as CDO_2_ < 3.8 mL O_2_/100 g/min. Neuro-ICU variables including intracranial pressure (ICP), pressure reactivity index, cerebral perfusion pressure (CPP), optimal CPP, and body temperature were assessed in association with the Xe-CT. The acute phase was divided into early phase (day 1–3) and vasospasm phase (day 4–14).

**Results:**

Of 148 patients, 27 had underwent a Xe-CT only in the early phase, 74 only in the vasospasm phase, and 47 patients in both phases. The patients exhibited cerebral hypoperfusion and poor CDO_2_ for medians of 15% and 30%, respectively, of the cortical brain areas in each patient. In multiple regressions, higher body temperature was associated with higher CBF and CDO_2_ in the early phase. In a similar regression for the vasospasm phase, younger age and longer pulse transit time (lower peripheral resistance) correlated with higher CBF and CDO_2_, whereas lower hematocrit only correlated with higher CBF but not with CDO_2_. ICP, CPP, and pressure reactivity index exhibited no independent association with CBF and CDO_2_. *R*^2^ of these regressions were below 0.3. Lower CBF and CDO_2_ in the early phase correlated with poor outcome, but this only held true for CDO_2_ in multiple regressions.

**Conclusions:**

Systemic and cerebral physiological variables exhibited a modest association with CBF and CDO_2_. Still, cerebral hypoperfusion and low CDO_2_ were common and low CDO_2_ was associated with poor outcome. Xe-CT imaging could be useful to help detect secondary brain injury not evident by high ICP and low CPP.

**Supplementary Information:**

The online version contains supplementary material available at 10.1007/s12028-022-01496-1.

## Introduction

Aneurysmal subarachnoid hemorrhage (aSAH) is a severe type of stroke that is associated with high mortality and severe neurological sequelae. Gradual development of cerebral vasospasm, ischemia, delayed ischemic neurologic deficits (DIND), and brain infarctions are feared complications following aSAH in the neurointensive care unit (neuro-ICU). Maintaining a sufficiently high cerebral blood flow (CBF) and cerebral oxygen delivery (CDO_2_) are key to avoid ischemic and hypoxic brain injury. Neuro-ICU management is based on monitoring and optimizing CBF and CDO_2_ surrogates. Specifically, mean arterial blood pressure (MAP) and intracranial pressure (ICP) together constitute the cerebral perfusion pressure (CPP) (CPP = MAP − ICP) [1, 2], which is targeted at more than 60 mm Hg to avoid cerebral ischemia [1, 2]. The CPP-therapy is based on fixed thresholds today, but this concept is flawed by not taking into account changes in cerebrovascular resistance and pressure autoregulation. A continuously dynamic optimal CPP (CPPopt) target, defined as the CPP with the concurrently best autoregulatory capacity [3], has been found to provide better brain tissue oxygenation [4] and energy metabolism [5] in patients with traumatic brain injury. CPPopt has also been found to better reflect CBF in aSAH [6] and could therefore be a better option than fixed CPP targets.

Arterial oxygen and carbon dioxide contents are also important for CBF and CDO_2_. Hyperventilation reduces partial pressure of carbon dioxide (pCO_2_) and leads to cerebral vasoconstriction and lower CBF, whereas hypercapnia may counteract ischemic CBF [7], increase brain tissue oxygenation [7], reduce DIND [8], and improve aSAH outcome [8]. Arterial hyperoxia may increase CDO_2_ and compensate for ischemic CBF and has shown promise in traumatic brain injury care [9, 10]. However, high partial pressure of oxygen (pO_2_) induces cerebral vasoconstriction [11] and reactive oxygen species [12] and is associated with cerebral vasospasm [13], DIND [14, 15], and unfavorable outcome[14, 15] in aSAH. Furthermore, arterial oxygen content (CaO_2_) is mainly dependent on the amount of oxygenated hemoglobin and a slight increase in the freely dissolved amount from hyperoxic pO_2_ only increases CaO_2_ to a small extent. On the other hand, hemoglobin has a major influence on CaO_2_, but higher values increase blood viscosity and reduce CBF [16]. Hemodilution with a lower hematocrit target is also a part of hemodilution, hypertension, and hypervolemia (HHH) therapy to increase CBF in cases of DIND [17], but because of the reduction in CaO_2_, less is known about the net effects on CDO_2_ [16–18].

Hence, neuro-ICU protocols with tightly regulated ICP/CPP and arterial content (pO_2_, pCO_2_, and hematocrit) aim to optimize CBF and CDO_2_, but there is limited knowledge to what extent these continuous multimodality monitoring variables can predict absolute CBF and CDO_2_ in aSAH. In the current study, the primary aim was to investigate the predictive role of these neuro-ICU measures on CBF and CDO_2_ at single measurements using Xenon-enhanced computed tomography (Xe-CT). Considering the theoretical effects of these variables but also the complex regulation of CBF, we hypothesized significant, but weak, associations between these variables and CBF and CDO_2_. The secondary aim was to determine the association among CBF and CDO_2_ with long-term clinical outcome. Considering the potential negative effects of cerebral ischemia and hypoxia on neuronal survival, and also the limited validity of a single measurement to affect outcome, we expected lower CBF and CDO_2_ only to be weakly yet significantly associated with worse neurological recovery.

## Materials and Methods

### Patients

Patients with aSAH, admitted to the Department of Neurosurgery at the Uppsala University Hospital, Sweden, between 2012 and 2020, were eligible for this study. All those 148 adult (age ≥ 18 years) patients who were intubated and mechanically ventilated with at least one Xe-CT scan within the first 14 days after aSAH were included in the study. Repeated Xe-CT was encouraged in all intubated and mechanically ventilated patients with aSAH, but not always possible due to intracranial hypertension, respiratory problems with high FiO_2_, and absence of staff familiar with the Xe-CT procedure.

### Treatment Protocol

Patients were treated in accordance with our standardized ICP- and CPP-oriented treatment protocol to avoid secondary insults, as described in detail in previous studies [1, 2]. Treatment goals were ICP ≤ 20 mm Hg, CPP ≥ 60 mm Hg, systolic blood pressure > 100 mm Hg, pO_2_ > 12 kPa, arterial glucose 5–10 mmol/L (mM), electrolytes within normal ranges, slight hypervolemia with zero fluid balance, hemoglobin ≥ 10 g/dL, and body temperature < 38 °C. The head of bed was generally elevated to 30°.

DIND was defined based on clinical observations, as new focal neurological symptoms and/or decreased level of consciousness when other causes, e.g. manifest infarction, intracerebral hematoma, and hydrocephalus, were excluded [19]. When DIND was ascertained, HHH-therapy was initiated, including supine position, colloid fluids (albumin and dextran solutions), hematocrit around 32%, and moderately elevated systolic blood pressure target more than 140 mm Hg [17]. Blood pressure targets were chiefly maintained with fluids, whereas inotropes/vasopressors were only used if systolic blood pressure/CPP still remained below the targeted thresholds. Dobutamine was used as the first line therapy for inotropic support and norepinephrine was considered a second line therapy for vasopressor support.

### Xe-CT and Calculation of CBF and Oxygen Delivery

The Xe-CT procedure were done in accordance with the principles described by Gur et al. [20] and Yonas et al. [21, 22] and has been described in detail in previous studies by our group [17, 23, 24]. The method is based on inhaled xenon gas being dissolved in blood and tissues, which then acts as a contrast agent for CT head scans and is used for CBF calculations [25, 26].

**Fig. 1:**
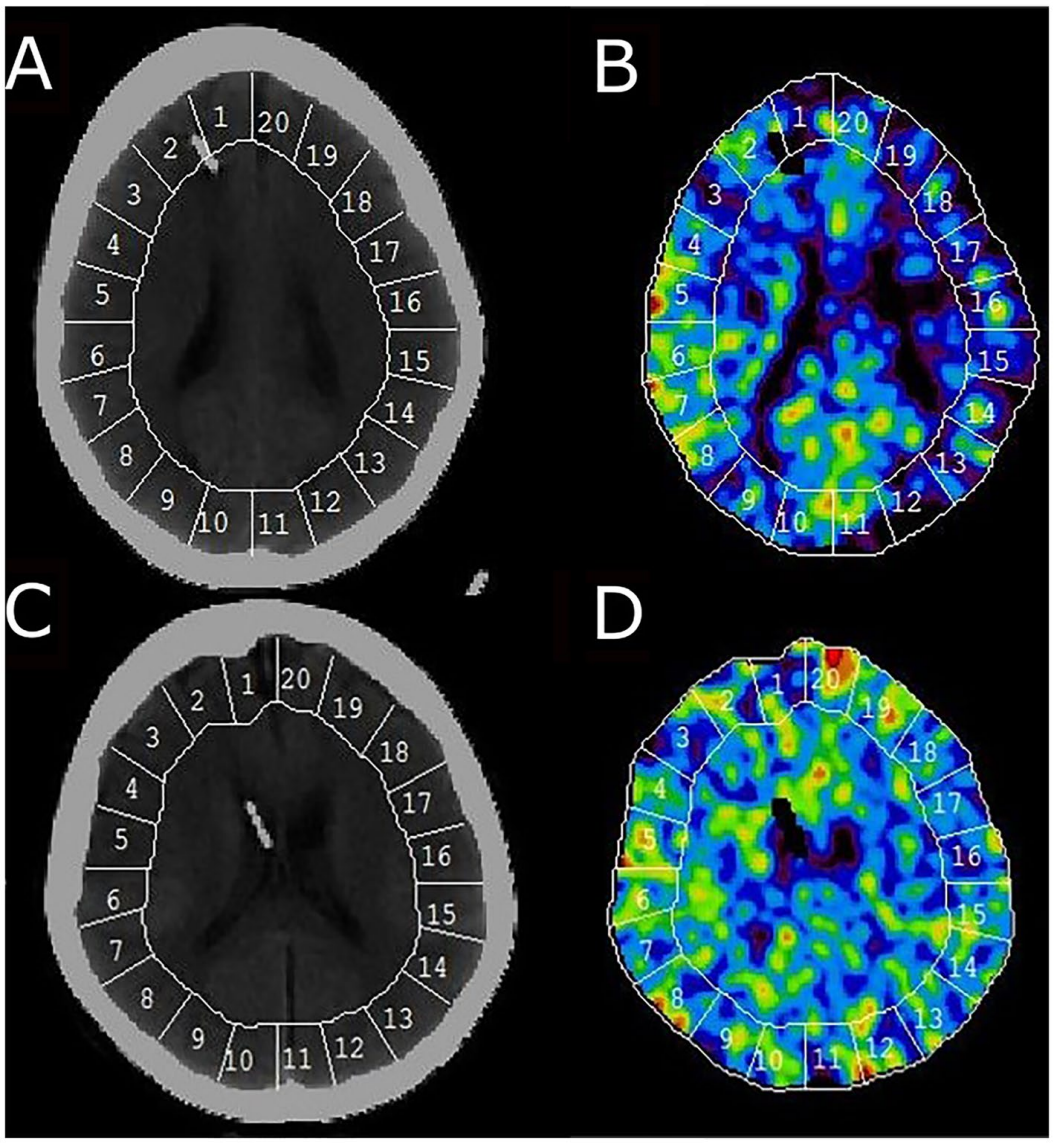
CBF measurements in two patients. The figure demonstrates two Xe-CT scans of two different patients. Patient 1 (**a**, **b**) exhibits extensive hypoperfusion particularly in the left hemisphere, whereas patient 2 (**c**, **d**) exhibits more normal CBF. CBF, cerebral blood flow, Xe-CT, xenon-enhanced computed tomography

The procedures were performed bedside in intubated and mechanically ventilated patients with aSAH in the neuro-ICU using mobile CT. The Enhancer 3000 xenon delivery system (Diversified Diagnostic Products Inc, Houston, TX) was connected to the ventilator and breathing circuit. Nonradioactive 28% Xe^131^ was administered to the breathing circuit for 4½ min, and CT scans synchronized to the xenon inhalation were obtained. Regional CBF in 20 cortical regions of interest (ROI; each around 350–450 mm^2^) of the CT image was calculated at four different levels of the brain (Fig. 1). The ROIs were visually inspected and single ROIs were occasionally removed in areas of hematomas or artifacts. Typically, three scan levels could be used for further calculations in each patient. Global cortical CBF in each individual patient was calculated as the mean value of all cortical ROIs (weighted by their ROI size). The burden of hypoperfusion and critical hypoperfusion was calculated as the percent of cortical brain areas with CBF < 20 mL/100 g/min and CBF < 10 mL/100 g/min, respectively [27]. Although the concept of global CBF and the burden of hypoperfusion and critical hypoperfusion were related, the latter two measures were more focal measures of the percent specific cortical brain areas that were vulnerable due to low CBF, whereas the global value could at least in theory still be adequate if one hemisphere was hyperemic and the other ischemic.

CDO_2_ was calculated based on the following formula [28] (adapted for kPa from mmHg):

$$\begin{array}{*{20}l} {{\text{CaO}}_{2} \left( {{\text{mL}}\;{\text{O}}_{2} {\text{/dL}}} \right) = {\text{Hemoglobin }}\left( {\text{g/dL}} \right) \times 1.34 \times {\text{SaO}}_{2} \times 10^{ - 2} + 22.5 \times 10^{ - 3} \times {\text{pO}}_{2} \left( {{\text{kPa}}} \right)} \hfill \\ {{\text{CDO}}_{2} \left( {{\text{mL}}\;{\text{O}}_{2} {/}100\;{\text{g/min}}} \right) = {\text{CBF}} \times {\text{CaO}}_{2} \times 10^{ - 2} } \hfill \\ \end{array}$$

The 1.34 constant reflects that the oxygen-carrying capacity in mL O_2_ per g/dL hemoglobin and the 22.5 constant the freely dissolved oxygen measured in mL O_2_ per pO_2_ kPa. The percent of brain areas with poor and severe CDO_2_ were calculated based on the definition of ischemic/critically ischemic CBF, respectively, in combination with a “normal” CaO_2_ including a hemoglobin at 14 g/dL, 100% SaO_2_, and a pO_2_ at 12 kPa [18]. Based on these numbers, poor and severe CDO_2_ were defined at < 3.8 mL O_2_/100 g/min and < 1.9 mL O_2_/100 g/min, respectively.

The Xe-CT results were analyzed in the early phase (day 1 to 3) and vasospasm phase (day 4 to 14), separately, to take into account pathophysiological temporal phases (early brain injury vs. vasospasm) and phases of treatment (focused on aneurysm occlusion in the early phase and avoiding DIND in the vasospasm phase).

### Data Acquisition

ICP was monitored with an external ventricular drain system (HanniSet, Xtrans, Smith Medical GmbH, Glasbrunn, Germany or VentrEX; Neuromedex, Hamburg, Germany) and occasionally with an intraparenchymal sensor device (Codman ICP Micro-Sensor; Codman & Shurtleff, Raynham, MA). Arterial blood pressure (ABP) was monitored in the radial artery at heart level. General arterial stiffness/systemic vascular resistance (SVR) is traditionally assessed as the carotid-femoral pulse wave velocity, but the carotid-radial transit has also been used [29–31]. In this study, to indirectly reflect SVR, pulse transit time (PTT) was calculated based on electrocardiography, ABP, and ICP. PTT was calculated as the time difference from every R spike of a heartbeat on the electrocardiography to when the ICP and radial ABP exceeded a threshold indicating systole, i.e. reflecting the difference in transit time from the heart to the brain (shorter) as compared to from the heart to the radial artery (longer) (Supplementary Figure 1). Pressure reactivity index (PRx) was calculated as the 5-min correlation of 10 s averages of ICP and MAP [32, 33]. CPPopt was calculated continuously as the CPP with the lowest PRx the last 4 h [3, 34]. Mean values of ICP, CPP, PRx, and CPPopt were calculated from 15 min before to 15 min after the Xe-CT (30 min duration). ∆CPPopt was defined as CPP–CPPopt. Physiological data were collected at 100 Hz using the Odin software [35]. PRx and CPPopt were calculated in retrospect and not used in clinical management of the patients. Arterial blood gases (ABGs) were extracted from the arterial line and analyzed on an ABL800 FLEX instrument (Radiometer, Copenhagen). ABGs were measured immediately before and after the Xe-CT and the mean value of these two measurements were used in the statistical analyses.

### Outcome

Clinical outcome was assessed according to the Extended Glasgow Outcome Scale (GOS-E) 12 months after ictus, by trained personnel using structured telephone interviews. GOS-E has eight categories of outcome that ranges from death (1) to upper good recovery (8) [36, 37]. Clinical outcome was dichotomized as favorable/unfavorable (GOS-E 5-8/1-4).

### Statistical Analysis

The analysis primarily (1) aimed to determine the explanatory variables for CBF and CDO_2_ and secondarily (2) the association of CBF and CDO_2_ with long-term clinical outcome.

The association among CBF (global cortical value and percent of hypoperfusion/critical hypoperfusion) and CDO_2_ (global cortical value and percent of poor/severe CDO_2_) indices with clinical and physiological variables was evaluated in the early phase (day 1–3) and the vasospasm phase (day 4–14) using the Spearman test. For patients with multiple Xe-CT measurements in each of the two phases, only the first scan of the phase was included. Multiple linear regression analyses were performed with CBF and CDO_2_, respectively, as the dependent variable, and the significant variables for any of the CBF and CDO_2_ indices in the univariate analyses were used as independent variables. Separate regressions were performed for the early phase and the vasospasm phase. The association among the CBF and CDO_2_ indices with clinical outcome (favorable/unfavorable) was analyzed with the Mann–Whitney *U*-test. Multiple logistic regressions for favorable outcome were also performed with age and World Federation of Neurosurgical Societies (WFNS) grade at admission as baseline variables and CBF and CDO_2_, respectively, in the early phase and the vasospasm phase as outcome predictors. Those with missing data were excluded from the analyses. A *p* value < 0.05 was considered statistically significant. The statistical analyses were done using SPSS version 28 (IBM Corp, Armonk, NY).

### Ethics

All procedures performed in the studies involving humans were in accordance with the ethical standards of the institutional and national research committee and with the 1964 Helsinki declaration and its later amendments. The study was approved by the regional ethical board (2010/138 and 2010/138/1) and the Swedish Ethical Review Authority (2020-05462).

## Results

### Patients, Admission Variables, Treatments, and Clinical Outcome

In this study, 148 patients were included (Supplementary Table 1). Twenty-seven patients had underwent a Xe-CT only in the early phase, 74 only in the vasospasm phase, and 47 patients in both phases. Fifty (34%) patients were men, and the mean age was 60 ± 12 years. At admission, median WFNS grade was 4 (interquartile range [IQR] 2–4) and median Fisher grade was 4 (IQR 3–4). The aneurysm was located in the anterior circulation in 125 (84%) cases and in the posterior circulation in 23 (16%) cases. One hundred twenty-two (82%) patients were treated for their aneurysm with endovascular embolization, 23 (16%) with clipping, 1 with both embolization and clipping, and 2 received no treatment. Fifty-two (35%) patients developed DIND and received HHH-therapy. Ten (7%) patients required thiopental, and 15 (10%) decompressive craniectomy due to high ICP. After 1 year, median GOS-E was 3 (IQR 3–5), 36 (26%) had a favorable recovery, and 23 (16%) were deceased.

### Systemic and Cerebral Physiological Variables During the CBF Imaging

The clinical and physiological data during the Xe-CT in the early phase and the vasospasm phase are described in Table 1. Median global cortical CBF was 30–35 mL/100 g/min and the percentage of cortical brain areas with hypoperfusion and critical hypoperfusion were approximately 15% and 1%, respectively, in both phases. CDO_2_ was around 5 mL O_2_/100 g/min, and the percent of poor and severe CDO_2_ were 30% and 3%, respectively.

**Table 1:** Systemic and cerebral physiological variables at the time point of xenon-enhanced computed tomography scans in the early phase and vasospasm phase

Parameter	Early phase (*n* = 74)	Vasospasm phase (*n* = 121)
pO_2_, median (IQR) (kPa)	13 (12 to 15)	13 (12 to 15)
pCO_2_, median (IQR) (kPa)	5.3 (4.9 to 5.7)	5.5 (5.2 to 5.9)
Hematocrit, median (IQR) (%)	34 (32 to 36)	33 (31 to 35)
HHH-therapy at the time of Xe-CT, *n* (%)	2 (3)	19 (16)
Vasopressor at the time of Xe-CT, *n* (%)	24 (32)	37 (31)
ICP, median (IQR) (mm Hg)	15 (13 to 18)	14 (10 to 16)
CPP, median (IQR) (mm Hg)	75 (69 to 80)	77 (70 to 85)
∆CPPopt, median (IQR) (mm Hg)	− 3 (− 10 to 7)	− 3 (− 8 to 6)
PRx (coefficient), median (IQR)	0.11 (0.02 to 0.23)	0.14 (0.01 to 0.27)
Body temperature, median (IQR) (°C)	37.5 (37.2 to 37.8)	38.1 (37.8 to 38.5)
PTT, median (IQR) (ms)	38 (28 to 48)	35 (24 to 43)
CBF, median (IQR) (mL/100 g/min)	33 (27 to 40)	35 (27 to 44)
Cerebral hypoperfusion, median (IQR) (%)	15 (6 to 39)	13 (3 to 28)
Critical cerebral hypoperfusion, median (IQR) (%)	1 (0 to 6)	0 (0 to 5)
CDO_2_, median (IQR) (mL O_2_/100 g/min)	5.1 (3.8 to 5.9)	5.0 (3.9 to 6.1)
Poor CDO_2_, median (IQR) (%)	31 (12 to 51)	29 (12 to 50)
Severe CDO_2_, median (IQR) (%)	3 (0 to 14)	3 (0 to 9)

Cerebral hypoperfusion and critical cerebral hypoperfusion were defined as the percentage of brain areas with CBF < 20 mL/100 g/min and and CBF < 10 mL/100 g/min, respectively. Poor and severe CDO_2_ were defined as CDO_2_ < 3.81 mL O_2_/100 g/min and CDO_2_ < 1.90 mL O_2_/100 g/min, respectively

CBF, cerebral blood flow, CDO_2_, cerebral delivery of oxygen, CPP, cerebral perfusion pressure, ∆CPPopt, CPP minus optimal cerebral perfusion pressure, HHH, hemodilution, hypertension, and hypervolemia, ICP, intracranial pressure, IQR, interquartile range, pO_2_, partial pressure of oxygen, PRx, pressure reactivity index, PTT, pulse transit time, Xe-CT, xenon-enhanced computed tomography

### CBF and Oxygen Delivery in the Early Phase and the Vasospasm Phase in Relation to Systemic and Cerebral Physiological Variables

In the early phase (Table 2, Supplementary Figure 2 and 3), lower CBF was associated with lower pCO_2_ and a lower body temperature. A higher percent of critical hypoperfusion was associated with higher PRx. A lower CDO_2_ was associated with older age, lower body temperature, and lower pCO_2_. A higher percent of brain areas with poor CDO_2_ was also associated with older age and lower body temperature, whereas a higher burden of severe CDO_2_ only correlated with lower body temperature.

**Table 2:** Clinical and physiological predictors of cerebral blood flow and hypoperfusion in the early phase and vasospasm phase: Spearman rank correlation analysis

Variables	Early phase (day 1–3)						Vasospasm phase (day 4–14)					
	CBF	Hypoperfusion	Critical hypoperfuson	CDO_2_	Poor CDO_2_	Severe CDO_2_	CBF	Hypoperfuson	Critical hypoperfusion	CDO_2_	Poor CDO_2_	Severe CDO_2_
Age	− 0.21	0.13	− 0.01	− 0.26*	0.26*	0.12	− 0.14	0.18*	0.13	− 0.24*	0.25**	0.26**
WFNS	− 0.02	0.02	0.03	− 0.04	− 0.04	0.04	− 0.11	− 0.01	− 0.10	− 0.05	− 0.01	− 0.06
Fisher	− 0.08	0.08	− 0.02	− 0.08	0.07	0.04	− 0.08	0.02	− 0.02	− 0.08	0.05	0.05
PTT	0.11	− 0.11	− 0.16	0.14	− 0.3	− 0.13	0.23*	0.15	− 0.27**	0.25**	− 0.21*	− 0.20*
pO_2_	0.09	− 0.10	− 0.09	0.10	− 0.09	− 0.08	− 0.01	0.00	0.11	0.08	− 0.02	0.08
pCO_2_	0.27*	− 0.22	− 0.18	0.24	− 0.18	− 0.19	0.10	− 0.02	0.10	0.06	− 0.08	0.03
Hematocrit	− 0.24	0.15	− 0.07	0.00	0.07	− 0.08	− 0.31***	0.26**	0.23*	− 0.04	0.05	0.18
ICP	0.21	− 0.19	− 0.08	0.17	− 0.17	− 0.06	0.09	− 0.05	0.04	0.05	− 0.04	− 0.03
CPP^a^	− 0.09	0.11	0.04	− 0.08	0.11	0.14	0.03	0.05	0.00	0.05	− 0.01	− 0.01
∆CPPopt	− 0.09	− 0.04	− 0.06	0.02	0.02	0.07	0.15	− 0.22*	− 0.21*	0.19	− 0.17	− 0.21*
PRx	− 0.01	0.12	0.25*	− 0.10	0.07	0.06	− 0.06	0.09	0.13	− 0.11	0.08	0.07
Body temperature	0.28*	− 0.22	− 0.23	0.34**	− 0.30*	− 0.31*	0.18	− 0.14	− 0.20*	0.19	− 0.16	− 0.23*

Hypoperfusion and critical hypoperfusion were defined as the percentage of brain areas with CBF < 20 mL/100 g/min and and CBF < 10 mL/100 g/min, respectively. Poor and severe CDO_2_ were defined as CDO_2_ < 3.81 mL O_2_/100 g/min and CDO_2_ < 1.90 mL O_2_/100 g/min, respectively. The table indicates the *r*-values of the Spearman correlation analyses

CBF, cerebral blood flow, CDO_2_, cerebral delivery of oxygen, CPP, cerebral perfusion pressure, CPPopt, optimal CPP, ∆CPPopt, CPP – CPPopt, HHH, hemodilution, hypertension, and hypervolemia, ICP, intracranial pressure, pO_2_, partial pressure of oxygen, pCO_2_, partial pressure of carbon dioxide, PRx, pressure reactivity index, PTT, pulse transit time, WFNS, World Federation of Neurosurgical Societies

*p < 0.05, **p < 0.01, ***p < 0.001. Bold values indicate statistical significance

^a^Exclusion of those patients with HHH therapy did not influence the results

In the vasospasm phase (Table 2, Supplementary Figure 4 and 5), lower CBF was associated with shorter PTT and higher hematocrit. A higher percentage of cerebral hypoperfusion correlated with older age, higher hematocrit, and CPP below CPPopt. A higher percentage of critical hypoperfusion was associated with shorter PTT, higher hematocrit, CPP below CPPopt, and lower body temperature. Furthermore, lower CDO_2_ was associated with older age and shorter PTT. Greater burden of poor CDO_2_ also correlated with older age and shorter PTT. Lastly, higher percentage of severe CDO_2_ was associated with older age, shorter PTT, CPP below CPPopt, and lower body temperature.

In multiple linear regression analyses of global cortical CBF in the early phase (Table 3), a higher body temperature was independently associated with higher CBF, whereas age, pCO_2_, and PRx showed no association with CBF. In a similar regression with CDO_2_ as the dependent variable, higher body temperature had a similar independent association, whereas the other variables were not associated with CDO_2_. In a multiple linear regression analyses of global cortical CBF in the vasospasm phase, younger age, higher PTT, and lower hematocrit were independently associated with higher CBF, whereas ∆CPPopt and body temperature were not associated with CBF. In a similar regression with CDO_2_ as the dependent variable, younger age and higher PTT were associated with higher CDO_2_, whereas hematocrit, ∆CPPopt, and body temperature were not associated with CDO_2_.* R*^2^ of these regressions were below 0.3.

**Table 3:** Predictors of cerebral blood flow and oxygen delivery in the early phase and the vasospasm phase: multiple linear regression analyses

Variables	Early phase			
	CBF		CDO_2_	
	*β*	*p*	*β*	*p*
Age	− 0.16	0.20	− 0.19	0.11
pCO_2_	0.14	0.25	0.12	0.32
PRx	0.00	0.99	− 0.09	0.46
Body temperature	0.31	**0.01**	0.34	**0.006**

Variables	Vasospasm phase			
	CBF		CDO_2_	
	*β*	*p*	*β*	*p*
Age	− 0.29	**0.003**	− 0.33	**0.002**
PTT	0.27	**0.006**	0.25	**0.02**
Hematocrit	− 0.39	**0.001**	− 0.06	0.57
∆CPPopt	0.10	0.32	0.07	0.49
Body temperature	0.14	0.16	0.12	0.25

Early phase: CBF, *R*^2^ = 0.17, ANOVA *p* value = 0.02, and *n* = 63. CDO2, *R*^2^ = 0.22, ANOVA *p* value = 0.005, and *n* = 63. Vasospasm phase: CBF, *R*^2^ = 0.30, ANOVA *p* value = 0.001, and *n* = 86. CDO_2_, *R*^2^ = 0.21, ANOVA *p* value = 0.003, and *n* = 80. Exclusion of those patients with HHH therapy did not influence the results

ANOVA, analysis of variance, CBF, cerebral blood flow, CDO_2_, cerebral delivery of oxygen, CPP, cerebral perfusion pressure, CPPopt, optimal CPP, ∆CPPopt = CPP − CPPopt, HHH, hypervolemia, hypertension, and hemodilution treatment, Rx, pressure reactivity index, pCO_2_, partial pressure of carbon dioxide, PTT, pulse transit time

Bold indicate statistical significance

### CBF and Oxygen Delivery in the Early Phase and the Vasospasm Phase in Relation to Clinical Outcome

**Fig. 2:**
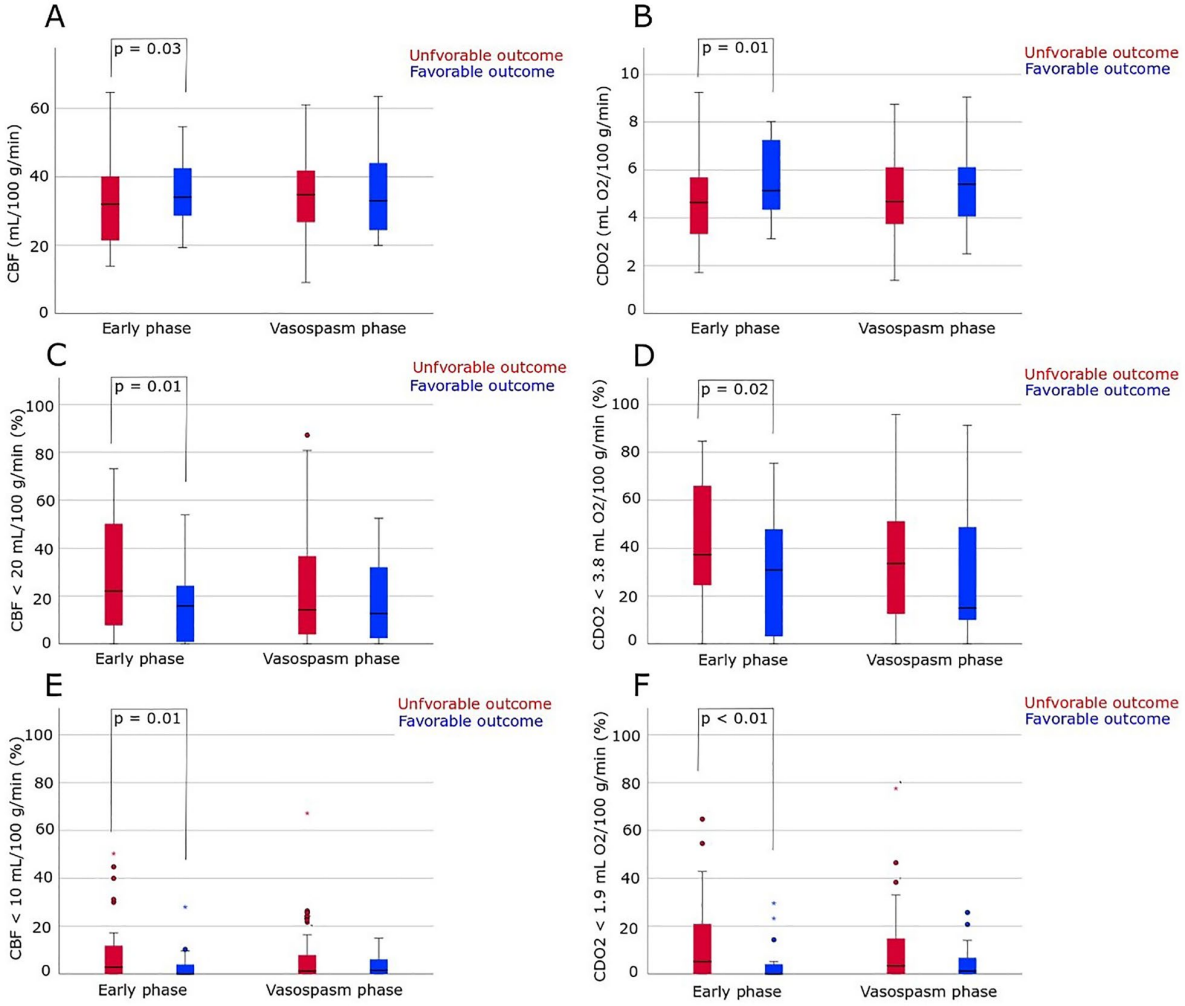
CBF and oxygen delivery in relation to clinical outcome after aneurysmal subarachnoid hemorrhage (**a**, **b**). A significant association between the CBF indices/CDO_2_ indices and clinical outcome was only present in the early phase. For those with unfavorable clinical outcome, global cortical CBF was lower (median 32 [IQR 22–40] vs. 36 [IQR 29–48] mL/100 g/min, *p* = 0.03) with a higher burden of hypoperfusion (median 21% [IQR 10–48] vs. 10% [IQR 2–20], *p* = 0.01) and critical hypoperfusion (median 3% [IQR 0–9] vs. 0% [IQR 0–3], *p* = 0.01). Similarly, those with unfavorable outcome had a lower CDO_2_ (median 5 [IQR 3–6] vs. 6 [IQR 4–7], *p* = 0.01) and higher burden of poor CDO_2_ (median 35 [IQR 25–63] vs. 11 [IQR 3–41], *p* = 0.02) and severe CDO_2_ (median 5 [IQR 0–20] vs. 0 [IQR 0–2], *p* = 0.001). However, no difference was found in the vasospasm phase between unfavorable and favorable outcome regarding CBF indices and CDO_2_ indices. Circles and stars indicate outliers (1.5–3 IQRs from the end of the box) and extreme outliers (more than 3 IQRs from the box), respectively. CBF, cerebral blood flow, CDO_2_, cerebral delivery of oxygen, ∆CPPopt, CPP − CPPopt, IQR, interquartile range, CBF, cerebral blood flow, CDO_2_, cerebral delivery of oxygen, CPP, cerebral perfusion pressure, CPPopt, optimal CPP, HHH, hypervolemia, hypertension, and hemodilution, ICP, intracranial pressure, PRx, pressure reactivity index, PTT, pulse transit time

A significant association between the CBF indices and CDO_2_ indices and clinical outcome was only present in the early phase (Fig. 2). For those with unfavorable clinical outcome, global cortical CBF was lower with a higher burden of cerebral hypoperfusion and critical hypoperfusion. Similarly, those with unfavorable outcome had a lower CDO_2_ and higher burden of poor CDO_2_ and severe CDO_2_ However, no difference was found in the vasospasm phase between unfavorable and favorable outcome regarding CBF indices and CDO_2_ indices. Hematocrit was not associated with clinical outcome in the early or the late phase (*p* > 0.05).

In multiple logistic regressions, higher CDO_2_ in the early phase was independently associated with favorable outcome after adjustment for age and WFNS grade (Table 4). This did not hold true in the vasospasm phase or for CBF if this variable replaced CDO_2_ in the regressions.

**Table 4:** CBF and CDO_2_ in the early phase and the vasospasm phase in relation to favorable outcome: multiple logistic outcome regression

Variables	Early phase (a)		Vasospasm phase (b)	
	OR (95% CI)	*p*	OR (95% CI)	*p*
Favorable outcome (1)				
Age	1.00 (0.95–1.05)	0.97	1.01 (0.97–1.05)	0.78
WFNS grade	0.50 (0.32–0.78)	0.002^a^	0.73 (0.50–1.05)	0.09
CBF	1.05 (1.00–1.10)	0.07	1.03 (0.98–1.07)	0.24
Favorable outcome (2)				
Age	1.00 (0.95–1.06)	0.94	1.00 (0.96–1.05)	0.87
WFNS grade	0.48 (0.30–0.77)	0.002^a^	0.69 (0.47–1.01)	0.06
CDO_2_	1.48 (1.05–2.09)	0.003^a^	1.20 (0.87–1.66)	0.27

Early phase, regression (1a) with CBF (Nagelkerke = 0.26, *n* = 72) and separate regression (2a) with CDO_2_ (Nagelkerke = 0.31, *n* = 69)

Vasospasm phase, regression (1b) with CBF (Nagelkerke = 0.07, *n* = 114) and separate regression (2b) with CDO_2_ (Nagelkerke = 0.08, *n* = 106)

CBF, cerebral blood flow; CDO_2_, cerebral delivery of oxygen; CI, confidence interval; OR, odds ratio; WFNS, World Federation of Neurosurgical Societies

^a^Statistical significance

## Discussion

In the current study including 148 patients with aSAH, the associations among neuro-ICUvariables with CBF and CDO_2_ were weak. Lower hematocrit correlated with higher CBF, but not with increased CDO_2_, indicating therapeutic limitations for the hemodilution component of HHH-therapy. Higher body temperature was associated with increased CBF and CDO_2_, but this likely reflected a compensatory increase to meet energy metabolic demand. ICP, CPP, and PRx only had modest associations with CBF and CDO_2_. Cerebral hypoperfusion and poor oxygen delivery were still frequent and particularly low CDO_2_ in the early phase was independently associated with unfavorable outcome. This indicates that common neuro-ICUvariables such as ICP and CPP were poor surrogates of CBF and CDO_2_, and this corroborates the indication for CBF imaging to better detect development of both global and focal secondary brain injury.

### Neurointensive Care Targets in Relation to CBF and Oxygen Delivery

In the current study, the association among neuro-ICU variables and CBF and CDO_2_ was limited. The absolute CPP was not significantly associated with CBF. This was to some extent expected, since CPP was kept within 60–100 mm Hg and cerebral autoregulation normally keeps CBF constant within these limits. However, cerebrovascular resistance may increase following vasospasm and most patients exhibited disturbed pressure autoregulation with PRx more than 0, predisposing for cerebral ischemia [38]. Considering the variable degree of such disturbances among patients, fixed CPP may not reliably rule out hypoperfusion/ischemia. Instead, more disturbed pressure autoregulation (higher PRx) and CPP below the autoregulatory CPPopt-target correlated better with cerebral hypoperfusion and poor CDO_2_, indicating that these measures may be superior to fixed/absolute CPP targets. This was consistent with previous studies by our group on similar but smaller cohorts of patients with aSAH [6, 39]. However, these associations were only found in the early phase after aSAH and did not hold true in multiple linear regressions. The role of CPPopt in aSAH has also been questioned because outcome studies have rather supported high fixed CPP targets than dynamic CPPopt targets [2]. Hence, much future work is still needed to determine whether CPPopt has a place in aSAH management. One major limitation include that PRx and CPPopt are global measures and may not be sensitive for focal vascular events. They may particularly be invalid in a scenario with asymmetrical vascular disturbances, such as when there is severe vasospasm in one hemisphere and vasodilation in the other. Another explanation is that the true CPPopt may be too high in the vasospasm phase to be fully explored on the *U*-shaped curve, which leads to an invalid underestimation.

Higher pCO_2_ correlated with higher CBF and was marginally associated with higher CDO_2_ in univariate, but not the multiple linear regression analyses in the early phase. This was in line with previous studies indicating that hypercapnia increases CBF and brain tissue oxygenation [7], reduces DIND [8], and improves clinical outcome in aSAH [8, 40, 41]. There was no association of pO_2_ with CBF and CDO_2_, despite previous findings supporting an association among hyperoxia with cerebral vasospasm [13], DIND [14, 15], and unfavorable outcome in aSAH [14, 15]. One explanation could be that both pCO_2_ and pO_2_ were tightly regulated at the time when the Xe-CT was performed, which reduced the chances to detect any effect on CBF and CDO_2_.

Furthermore, lower hematocrit was strongly associated with higher CBF and lower percent of hypoperfusion and critical hypoperfusion in the vasospasm phase. However, this did not translate into better CDO_2_, as a consequence of the corresponding reduction in CaO_2_ from the lower hematocrit value. The increase in CBF may be related to a reduction in blood viscosity or successful hypervolemia treatment leading to both higher intravascular volume and CBF, but others suggest that it is solely a partially compensatory increase in CBF to meet CDO_2_ demand [16]. Nevertheless, despite the lack of improvement in CDO_2_, higher CBF from hemodilution could improve cerebral microcirculation, glucose delivery, and clearance, but this argument is only speculation. Increased glucose delivery could be particularly important considering that mitochondrial dysfunction with a reduced capacity to use oxygen is common after aSAH [42]. However, others have rather discussed the possibility to improve CDO_2_ by means of hypervolemia and higher hematocrit using red blood cell transfusion [43]. Overall, this still questions the therapeutic effect of hemodilution in HHH therapy. Previous experience from our group is that HHH therapy for DIND increases CBF and is neither associated with a worsening nor an improvement in cerebral energy metabolism, as assessed with a microdialysis [24]. The results could either be interpreted as that a more severe energy metabolic worsening was prevented or that it had no beneficial effect on the brain. It is also difficult to isolate the hemodilution component from the HHH therapy. Altogether, multimodality monitoring studies combining CBF imaging, brain tissue oxygenation, and microdialysis could further elucidate the cerebral effects of different components of HHH in aSAH care.

Increased body temperature was associated with higher CBF and CDO_2_. This was likely a compensatory mechanism to meet increased energy metabolic demand [44]. This increase is often usually only partially compensatory to energy demand and is not a viable treatment option to counteract ischemia, since hyperthermia has previously been associated with DIND, and worse clinical outcome in aSAH [2, 45].

Older age was a clinical variable associated with lower CBF and particularly lower CDO_2_. Although older patients are not more prone to develop vasospasm or DIND [46], they are fragile and often exhibit a combination of arterial hypotension and hypoxemic insults [47]. This could explain the increased susceptibility for secondary ischemic/hypoxic brain injury as was evident in our study. Furthermore, increased PTT was independently associated with higher CBF and CDO_2_. This suggests an association between a higher SVR and higher risk of cerebral hypoperfusion and hypoxia. The underlying factor for SVR could be arterial stiffness from atherosclerosis, but also from stress-induced and vasopressor-induced vasoconstriction. This also suggests that an attempt to increase MAP/CPP by vasopressors, which potentially increases SVR/PTT, may exert a negative effect on CBF and CDO_2_, whereas measures made to increase intravascular volume and cardiac output might be more favorable approaches.

Altogether, the associations among neuro-ICU variables with CBF and CDO_2_ were weak, but cerebral hypoperfusion and poor CDO_2_ were relatively frequent at 15% and 30%, respectively. This supports the role of Xe-CT imaging to detect secondary brain injury development, not evident by standard neuro-ICU variables such as high ICP and low CPP. Also focal, continuous CBF monitoring such as thermal dilution methods could be of value and may be explored in future studies. Although the associations between neuro-ICU variables and CBF/CDO_2_ were weak when evaluated as absolute values, interventions that change systemic variables such as CPP augmentation or pCO_2_ elevation would be expected to at least exert some effect on CBF/CDO_2_. However, our study was not designed to determine whether any specific treatment intervention would be superior to another in this scenario.

### CBF and Oxygen Delivery in Relation to Clinical Outcome

Low CBF and CDO_2_ in the early phase were associated with unfavorable outcome, but this did only hold true for CDO_2_ in multiple logistic regressions and not in the vasospasm phase. The association among CBF and CDO_2_ with outcome was hence stronger in the early phase than the vasospasm phase. One explanation could be that early CBF disturbances also predicts a worse neuro-ICU course, as early cerebral hypoperfusion tends to persist in the late course [23] and has been associated with DIND [48]. Apparently, early assessment of CBF and CDO_2_ may be valuable to identify patients at risk of having poor outcome with the intention to individualize their treatment and change their destined clinical course. Another explanation for the association between low CBF and CDO_2_ and worse outcome could be that the former two were metabolically downregulated following a more severe brain injury, although this was taken into account to some extent by adjusting for WFNS grade. However, overall, the weak association between CBF and CDO_2_ and clinical outcome was expected, considering that the measurement only reflected a snapshot of the acute phase after aSAH.

### Limitations

First, the associations between systemic and cerebral physiological variables and CBF and CDO_2_ were generally weak. This may be explained by that these latter two variables are regulated by a complex plethora of mechanisms that interact with each other rather than being determined by one single variable. It was also evident that severely deranged physiological variables were infrequent, which likely reduced any chance to detect an effect of these variables on CBF and CDO_2_. Second, PTT, ICP, CPP, CPPopt, PRx, and body temperature were calculated for 30 min centered on the time point of the Xe-CT. The reason for such a long interval was to get a stable value, particularly for CPPopt and PRx. However, if the physiological variables were calculated only for 1 min at the time point of the Xe-CT (data not shown), similar, but slightly weaker associations were found for CPPopt/PRx and CBF and CDO_2_. Third, the study group was highly selected to be patients in a bad clinical situation, who were intubated and with specific requirements on the monitoring for inclusion in the study. This, together with the limited number of individuals could explain why some tests did not reach statistical significance. Fourth, reduced CBF may be explained a reduction in energy metabolic demand by, e.g., concurrent brain injury, hypothermia and sedation rather than ischemia. However, the burden of hypoperfusion and critical hypoperfusion was calculated as the percent of cortical brain areas with CBF < 20 mL/100 g/min and CBF < 10 mL/100 g/min, respectively. These measures rather reflect the proportion of focal hypoperfusion rather than global changes related to sedation, etc. Fifth, including the patients treated with DIND may have influenced the overall results but excluding the patients with DIND from the univariate correlation analyses did not affect the associations. Sixth, DIND was diagnosed based on clinical ground by experienced clinicians, although it was possible that false positives and negatives (silent brain infarctions) occurred to some extent. Seventh, ABP was measured at heart level which overestimates CPP to some extent when the head of the bed is elevated but this did not affect the calculation of ∆CPPopt because it is the difference between CPP and CPPopt. The results may have been confounded by that a smaller group of patients receiving HHH therapy was placed in supine position but the results did not change when those patients were excluded from the analysis.

## Conclusions

Older patients with increased SVR were at increased risk of low CBF and oxygen delivery. A lower hematocrit correlated with higher CBF but not with increased oxygen delivery, which suggests a limited beneficial cerebral effect from hemodilution. Higher body temperature was associated with increased CBF and oxygen delivery but was more likely a consequence of increased energy metabolic demand. ICP, CPP, and pressure reactivity only had modest associations with CBF and oxygen delivery. Cerebral hypoperfusion and poor oxygen delivery were still common, which indicates that common neurointensive care variables were poor surrogates of these targets, and this corroborates the indication for multimodality monitoring, including CBF imaging, to better detect the development of secondary brain injury.

## Supplementary Information

Below is the link to the electronic supplementary material.

Supplementary file1 (DOCX 71 kb)

Supplementary file2 (DOCX 331 kb)

Supplementary file3 (DOCX 252 kb)

Supplementary file4 (DOCX 337 kb)

Supplementary file5 (DOCX 262 kb)

Supplementary file6 (DOCX 12 kb)
