# Correction to: Cerebral Blood Flow and Oxygen Delivery in Aneurysmal Subarachnoid Hemorrhage: Relation to Neurointensive Care Targets

**DOI:** 10.1007/s12028-022-01528-w

**Published:** 2022-05-23

**Authors:** Teodor Svedung Wettervik, Henrik Engquist, Anders Hånell, Timothy Howells, Elham Rostami, Elisabeth Ronne-Engström, Anders Lewén, Per Enblad

**Affiliations:** 1 Section of Neurosurgery, Department of Neuroscience, Uppsala University, Uppsala, Sweden; 2 Department of Surgical Sciences/Anesthesia and Intensive Care, Uppsala University, Uppsala, Sweden

## Correction to: Neurocrit Care 10.1007/s12028-022-01496-1

This article was updated to correct a typo written in the “Discussion”:

"Low CBF and CDO2 in the early phase were associated with unfavorable outcome, but this did only hold true for CBF in multiple logistic regressions and not in the vasospasm phase."

but is supposed to be:

"Low CBF and CDO2 in the early phase were associated with unfavorable outcome, but this did only hold true for ***CDO2*** in multiple logistic regressions and not in the vasospasm phase."

