# Supplementary material for: Cerebral Blood Flow and Oxygen Delivery in Aneurysmal Subarachnoid Hemorrhage: Relation to Neurointensive Care Targets
Source: Neurocrit Care. 2022 Apr 21;37(1):281–92. doi: 10.1007/s12028-022-01496-1 (PMC9283361; doi:10.1007/s12028-022-01496-1)
Supplement: Supplementary file 1 — Supplementary file1 (DOCX 71 kb) [file 12028_2022_1496_MOESM1_ESM.docx]

**Supplementary Figure 1. Calculation of the pulse transit time**

**
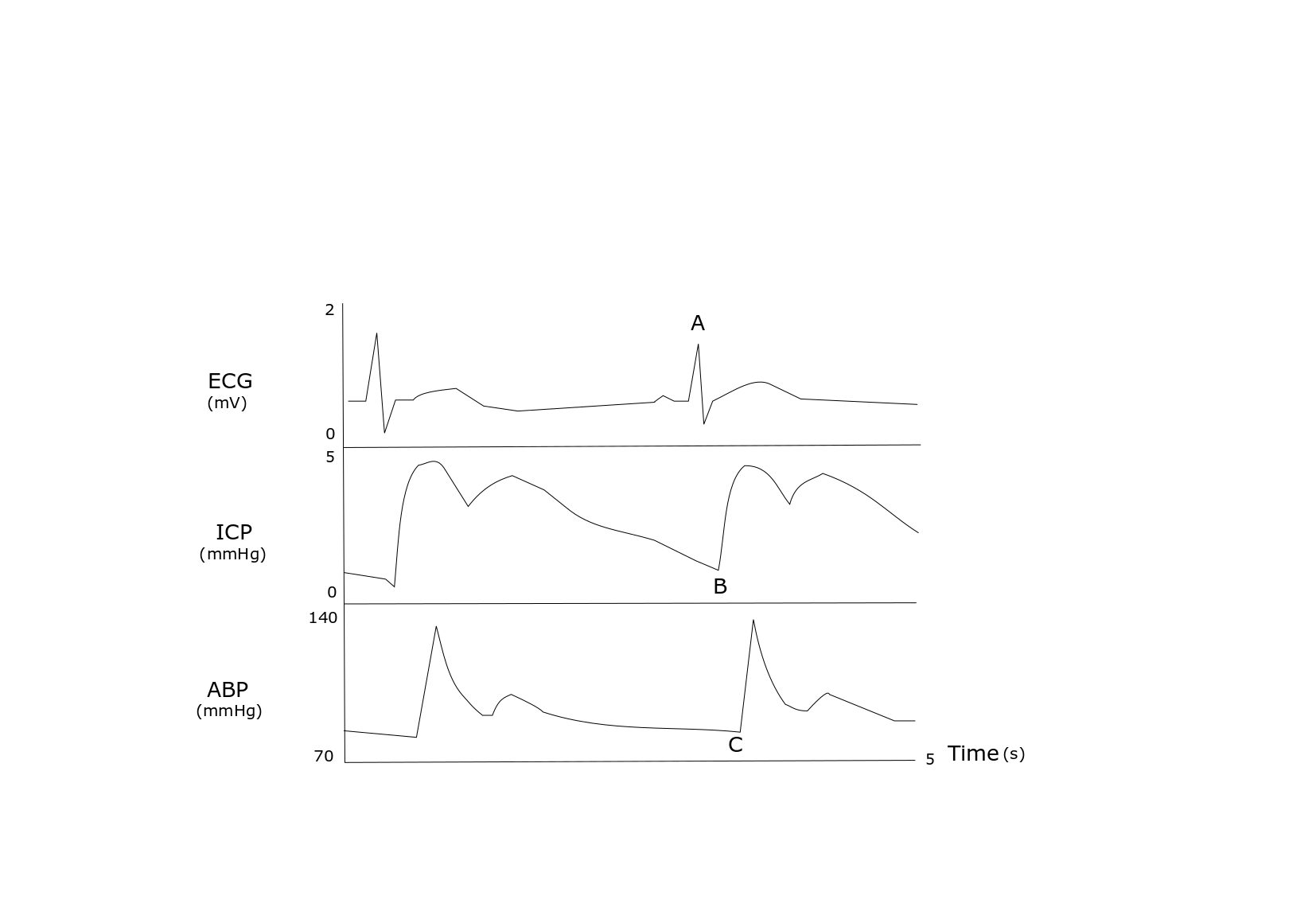
**

The figure illustrates the calculation of PTT. **A** indicates the R spike during a heartbeat, registered on the ECG. **B** indicates the upstick of the systolic ICP pulse pressure wave following the cardiac beat **A**. **C** indicates the upstick of the arterial blood pressure wave in the radial artery following **A**. The PTT is defined as the difference between the pulse transit time from the heart to the radial artery and the pulse transit time from the heart to the brain, i.e. the horizontal distance between **B** and **C** in time (ms). Hence, PTT is calculated as the difference in time from the R spike (**A**) in a heartbeat to the systolic intracranial and arterial radial pressure wave, respectively. As the arterial distance to the intracranial space is shorter than to the radial artery, the intracranial pressure wave precedes the radial pressure wave. Lower PTT values indicate increased SVR.

ECG = Electrocardiography. PTT = Pulse transit time. SVR = Systemic vascular resistance.
