# Supplementary material for: Cerebral Blood Flow and Oxygen Delivery in Aneurysmal Subarachnoid Hemorrhage: Relation to Neurointensive Care Targets
Source: Neurocrit Care. 2022 Apr 21;37(1):281–92. doi: 10.1007/s12028-022-01496-1 (PMC9283361; doi:10.1007/s12028-022-01496-1)
Supplement: Supplementary file 2 — Supplementary file2 (DOCX 331 kb) [file 12028_2022_1496_MOESM2_ESM.docx]

**Supplementary Figure 2. Systemic and cerebral physiological variables in relation to CBF and CDO_2_ in the early phase**

**
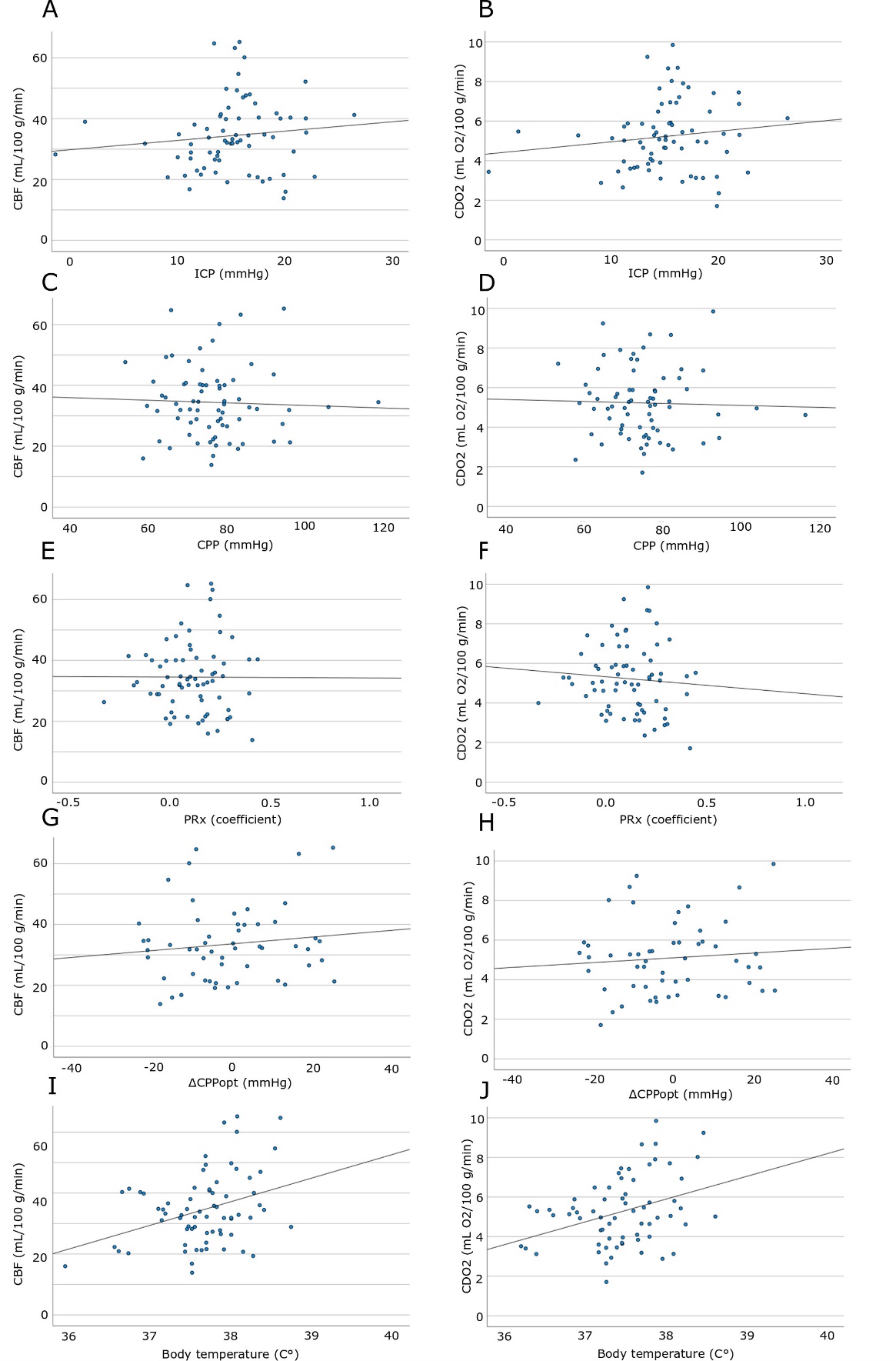
**

The figure demonstrates the associations among CBF and CDO_2_ with ICP (1A-B), CPP (1C-D), PRx (1E-F), ∆CPPopt (1G-H), and body temperature (1I-J) in the vasospasm phase. Higher body temperature correlated (Spearman) with higher CBF (r = 0.28, p < 0.05) and higher CDO_2_ (r = 0.34, p < 0.01). There was no association among any of the physiological variables with global CBF and CDO_2_.

CBF = Cerebral blood flow. CDO_2_ = Cerebral delivery of oxygen. CPP = Cerebral perfusion pressure. CPPopt = Optimal CPP. ICP = Intracranial pressure. PRx = Pressure reactivity index. ∆CPPopt = CPP-CPPopt
