# Supplementary material for: Cerebral Blood Flow and Oxygen Delivery in Aneurysmal Subarachnoid Hemorrhage: Relation to Neurointensive Care Targets
Source: Neurocrit Care. 2022 Apr 21;37(1):281–92. doi: 10.1007/s12028-022-01496-1 (PMC9283361; doi:10.1007/s12028-022-01496-1)
Supplement: Supplementary file 4 — Supplementary file4 (DOCX 337 kb) [file 12028_2022_1496_MOESM4_ESM.docx]

**Supplementary figure 4A-J. Systemic and cerebral physiological variables in relation to CBF and CDO_2_ in the vasospasm phase**


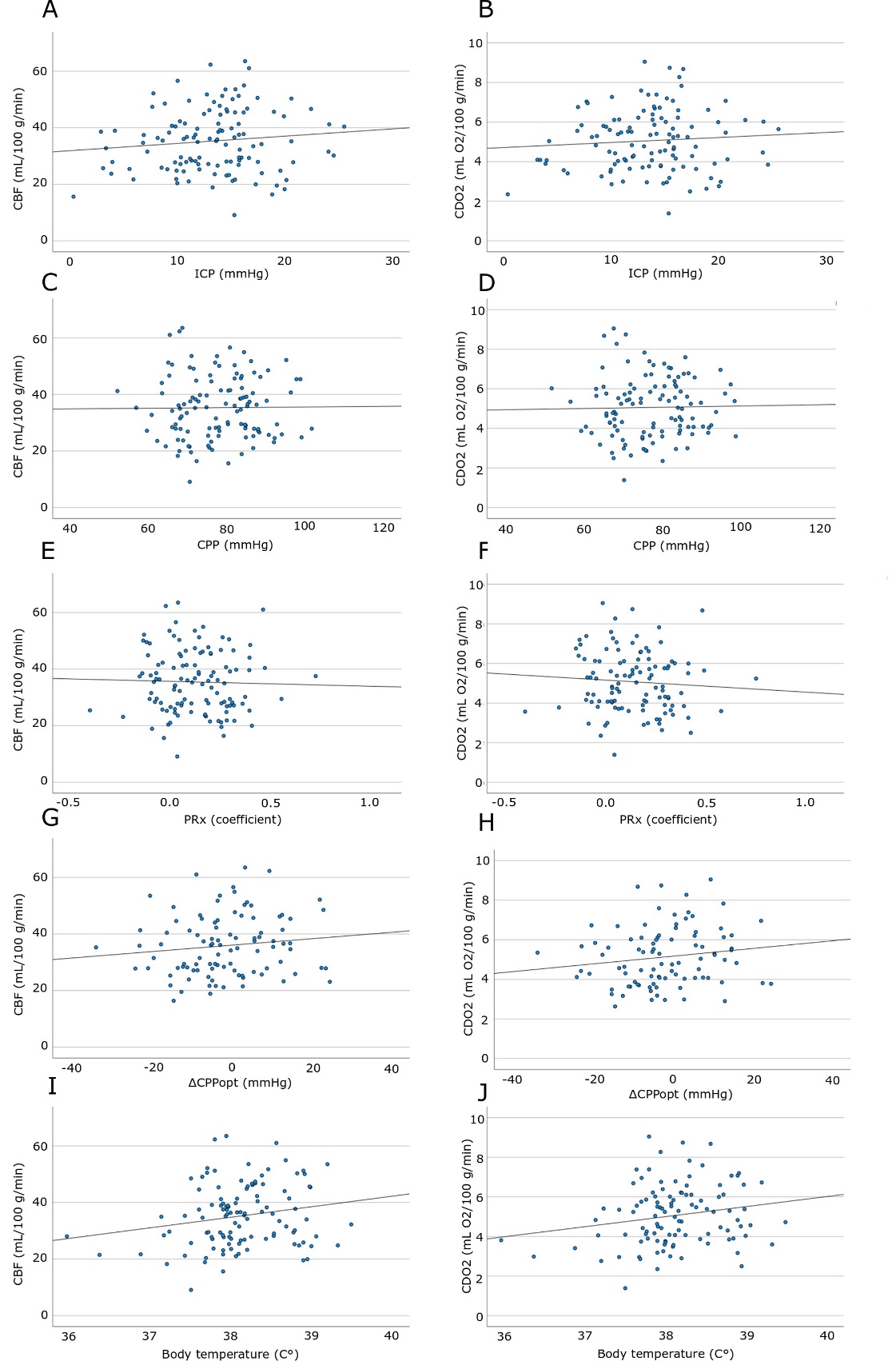


The figure demonstrates the associations among CBF and CDO_2_ with ICP (3A-B), CPP (3C-D), PRx (3E-F), ∆CPPopt (3G-H), and body temperature (3I-J) in the vasospasm phase. There was no association among any of the physiological variables with global CBF and CDO_2_.

CBF = Cerebral blood flow. CDO_2_ = Cerebral delivery of oxygen. CPP = Cerebral perfusion pressure. CPPopt = Optimal CPP. ICP = Intracranial pressure. PRx = Pressure reactivity index. ∆CPPopt = CPP-CPPopt
