# Supplementary material for: Cerebral Blood Flow and Oxygen Delivery in Aneurysmal Subarachnoid Hemorrhage: Relation to Neurointensive Care Targets
Source: Neurocrit Care. 2022 Apr 21;37(1):281–92. doi: 10.1007/s12028-022-01496-1 (PMC9283361; doi:10.1007/s12028-022-01496-1)
Supplement: Supplementary file 5 — Supplementary file5 (DOCX 262 kb) [file 12028_2022_1496_MOESM5_ESM.docx]

**Supplementary figure 5A-F. Arterial content in relation to CBF and CDO_2_ in the vasospasm phase**


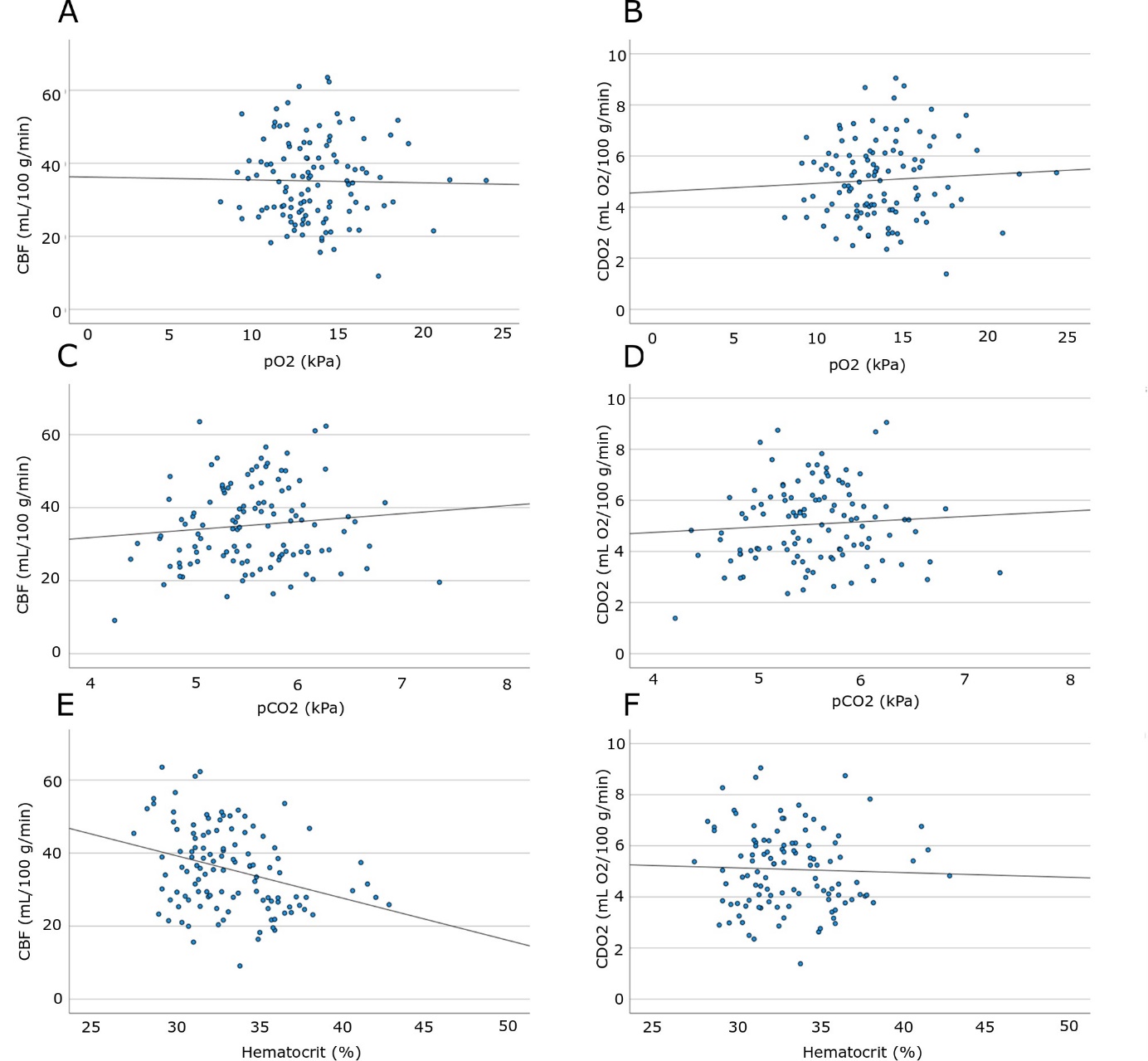


The figure demonstrates the associations among CBF and CDO_2_ with pO_2_ (4A-B), pCO_2_ (4C-D), and hematocrit (4E-F) in the vasospasm phase. Lower hematocrit was significantly associated (Spearman) with higher CBF (r = -0.31, p < 0.001), but not with CDO_2_. There were no association among pO_2_ and pCO_2_ with CBF and CDO_2_.

CBF = Cerebral blood flow. CDO_2_ = Cerebral delivery of oxygen.
