# Supplementary material for: Cerebral Blood Flow and Oxygen Delivery in Aneurysmal Subarachnoid Hemorrhage: Relation to Neurointensive Care Targets
Source: Neurocrit Care. 2022 Apr 21;37(1):281–92. doi: 10.1007/s12028-022-01496-1 (PMC9283361; doi:10.1007/s12028-022-01496-1)
Supplement: Supplementary file 6 — Supplementary file6 (DOCX 12 kb) [file 12028_2022_1496_MOESM6_ESM.docx]

**Supplementary Table 1. Demography, admission status, treatments, and clinical outcome**

| Patients, n (%) | 148 (100%) |
| --- | --- |
| Age (years), mean (±SD) | 60 ± 12 |
| Sex (male/female), n (%) | 50/98 (34/66%) |
| WFNS grade, median (IQR) | 4 (2-4) |
| Pupillary abnormality, n (%) | 3 (2%) |
| Fisher grade, median (IQR) | 4 (3-4) |
| Aneurysm location (anterior/posterior), n (%) | 125/23 (84/16%) |
| Aneurysm treatment (none/embolization/clipping/both), n (%) | 2/122/23/1 (1/82/16/1%) |
| DIND, n (%) | 52 (35%) |
| ICP monitor (none/EVD/Codman/Both), n (%) | 1/141/0/6 (1/95/0/4%) |
| Thiopental, n (%) | 10 (7%) |
| Decompressive craniectomy, n (%) | 15 (10%) |
| GOS-E*, median (IQR) | 3 (3-5) |
| Favorable outcome*, n (%) | 36/104 (26/74%) |
| Mortality* | 23/117 (16/84%) |

*8 patients with missing outcome data.

DIND = Delayed ischemic neurological deficit. EVD = External ventricular drain. GOS-E = Glasgow Outcome Scale-Extended. ICP = Intracranial pressure. IQR = Interquartile range. SD = Standard deviation. WFNS = World Federation of Neurosurgical Societies.
